# Production of Palmitoleic and Linoleic Acid in Oleaginous and Nonoleaginous Yeast Biomass

**DOI:** 10.1155/2016/7583684

**Published:** 2016-02-28

**Authors:** Irena Kolouchová, Olga Maťátková, Karel Sigler, Jan Masák, Tomáš Řezanka

**Affiliations:** ^1^Department of Biotechnology, University of Chemistry and Technology Prague, Technická 5, 166 28 Prague, Czech Republic; ^2^Institute of Microbiology, CAS, Vídeňská 1083, 142 20 Prague, Czech Republic

## Abstract

We investigated the possibility of utilizing both oleaginous yeast species accumulating large amounts of lipids (*Yarrowia lipolytica, Rhodotorula glutinis, Trichosporon cutaneum, *and* Candida *sp.) and traditional biotechnological nonoleaginous ones (*Kluyveromyces polysporus, Torulaspora delbrueckii, *and* Saccharomyces cerevisiae*) as potential producers of dietetically important major fatty acids. The main objective was to examine the cultivation conditions that would induce a high ratio of dietary fatty acids and biomass. Though genus-dependent, the type of nitrogen source had a higher influence on biomass yield than the C/N ratio. The nitrogen source leading to the highest lipid accumulation was potassium nitrate, followed by ammonium sulfate, which is an ideal nitrogen source supporting, in both oleaginous and nonoleaginous species, sufficient biomass growth with concomitantly increased lipid accumulation. All yeast strains displayed high (70–90%) content of unsaturated fatty acids in total cell lipids. The content of dietary fatty acids of interest, namely, palmitoleic acid and linoleic acid, reached in* Kluyveromyces *and* Trichosporon *strains over 50% of total fatty acids and the highest yield, over 280 mg per g of dry cell weight of these fatty acids, was observed in* Trichosporon *with ammonium sulfate as nitrogen source at C/N ratio 70.

## 1. Introduction

Microorganisms produce lipids in standard quality; the waste biomass and byproducts are biodegradable and nontoxic [1–5]. All types of microorganisms produce lipids but they can be differentiated by the amount produced: nonoleaginous strains (the majority of microorganisms, many technologically employed strains, e.g.,* Saccharomyces* sp.) do not accumulate lipids in high quantity while oleaginous strains accumulate more than 20% lipids in dry cell weight and can be exploited for production of fatty acid methyl esters, that is, biodiesel [6]. Oleaginicity is not clade specific: oleaginous and nonoleaginous species are known in the same genus [7, 8].

Yeasts appear to be the most easily exploitable microorganism due to their fast lipid accumulation, relatively high biomass yield, and unicellular character [9, 10]. These properties might be advantageous for subsequent lipid isolation and processing and following biodiesel production. At the present costs of fossil fuels, the production of microbial-derived biofuels is not yet a promising alternative. However, the possibility is intensely investigated and publications on the many aspects of this scientific topic are available [1, 7, 11]. The technoeconomical evaluation of the process has been published [12] and it has been demonstrated that the overall cost of the process is principally due to the cost of the fermentation (e.g., aeration and agitation) in large scale and the starting material does not play a material role.

Numerous studies have reported oil content and/or fatty acid composition of yeasts [10, 13, 14], but these studies are often describing only a few yeast species. Yeast lipids are mostly present in the cells in the form of triacylglycerols; some of these lipids are constituted by monounsaturated and polyunsaturated fatty acids (PUFA) [15], whose production might be exploitable due to the ability of these microorganisms to synthesize in high quantities lipids rarely found in the plant or animal kingdom (i.e., lipids presenting composition similarities with the cocoa butter or other exotic fats). Essential fatty acids, (n-3 and n-6 series) are not usually produced by yeast strains [16], but other unsaturated fatty acids of interest, such as palmitoleic and linoleic acid, can form a substantial part of microbial lipids [17]. The lipid amount and composition are highly dependent on substrate (glucose, volatile fatty acid (VFA), and derivative animal fat) [18–21], nutrient availability (N, P, and S), temperature, pH, cultivation time, and setup (batch, fed batch) [2, 22, 23]. For industrial-scale production and application, it is necessary to find a balance between the maximum lipid production (as influenced by the above factors) and type and price of substrate and economical balance of the production, including the financial demands of subsequent lipid isolation [24–27]. Optimization of lipid production may involve genetic manipulation and targeted microbial alteration aiming at, for example, increasing the content of omega-3 and omega-6 unsaturated fatty acids [19, 20]. PUFA are essential fatty acids and, as humans cannot synthetize them, they must be ingested. Their deficiency leads to, for example, impaired arachidonic acid biosynthesis and subsequent synthesis of prostaglandins and results in imperfect growth of skeletal muscle tissue and hair loss [28, 29].

Palmitoleic acid is a monounsaturated fatty acid that belongs among beneficial fatty acids. It has been shown to have many important health benefits and therefore has broad applications in medicine and cosmetics [17, 30]. Its limited availability makes it a target of studies focused on health supplements production. Linoleic acid (18 : 2) is a polyunsaturated n-6 fatty acid that participates in the fat metabolism and also has been also proven to have positive effects in the treatment of many diseases. Both fatty acids are therefore the object of this study as to the possibility of their production by microorganisms.

The main factor in lipid accumulation is the C/N ratio of the nutrient medium. High C/N ratio leads to high cellular lipid content, but at ratios higher than 100, the amount of produced lipids decreases, when glucose was used as carbon source [23, 31, 32]. Higher lipid content in the cells is often associated with lower biomass yield. This phenomenon is highly dependent on the type of nitrogen source; however, besides the maximum lipid production, one must in this context consider also the economic perspective, as the prices of inorganic nitrogen sources such as ammonium chloride are much lower than those of complex sources such as yeast extract and peptone [33].

We employed seven yeast strains, both oleaginous and nonoleaginous ones that are often used in biotechnological applications and can be obtained in high quantities as waste biomass and/or are able to produce high biomass yields. This work is focused on the influence of nitrogen source on the growth, total lipid accumulation, and unsaturated fatty acids (namely, linoleic and palmitoleic acid) of seven yeast strains, both commonly and less commonly employed for microbial lipid production (*Yarrowia lipolytica, Rhodotorula glutinis, Trichosporon cutaneum, Candida* sp.,* Torulaspora delbrueckii, Kluyveromyces polysporus,* and* Saccharomyces cerevisiae*).

## 2. Materials and Methods

### 2.1. Microorganisms

The yeast strains used in the present study were* Candida* sp. DBM 2163;* Kluyveromyces polysporus* DBM 2171;* Rhodotorula glutinis* CCY 20-2-20;* Saccharomyces cerevisiae* DBM 2115;* Torulaspora delbrueckii* DBM 39;* Trichosporon cutaneum* CCY 30-5-10;* Yarrowia lipolytica* CCY 29-26-36 supplied by Culture Collection of Yeast, Institute of Chemistry, Slovak Academy of Sciences, Bratislava, and by Collection of Yeasts and Industrial Microorganisms of University of Chemistry and Technology, Prague. For long term storage, the stock cultures were maintained in 20% glycerol at −60°C. Malt extract agar (23 g/L, pH 7) was employed for short term storage.

### 2.2. Cultivation Conditions

The precultures of yeast strains were cultivated in 200 mL of YPD medium (20 g/L peptone, 10 g/L yeast extract, and 20 g/L glucose, initial pH 6.0) in Erlenmeyer flasks on a rotary shaker at 150 rpm at 28°C to the late exponential growth phase.

For lipid production, 200 mL of mineral medium in 500 mL Erlenmeyer flasks was inoculated with 10 mL of preculture to a final concentration of OD_600_ 0.2 and incubated on a rotary shaker at 150 rpm and 28°C. Mineral medium composition was (g/L) Na_2_HPO_4_·12H_2_O, 5; KH_2_PO_4_, 7; trace element solution 1 mL (MnCl_2_·4H_2_O, 20; FeSO_4_·7H_2_O, 1; Na_2_MoO_4_·2H_2_O, 1; CaCl_2_·2H_2_O, 20), pH 6.5. Glucose was added as carbon source to the concentration 30 g/L and nitrogen source was supplemented so as to achieve different C/N ratios.

Growth characteristics were determined in the above yeast strains cultivated on different C/N ratios and with different N sources ((NH_4_)_2_SO_4_, KNO_3_, urea, and (NH_4_)NO_3_) in the Bioscreen C (Labsystems, Finland) device in microtiter plates. Cultivation temperature was 30°C, and optical density was determined every 2 h. All experiments were performed five times. For lipid content and biomass yield determination, the yeasts were cultivated in 100 mL mineral medium in Erlenmeyer flasks (3 mL inoculum) on orbital rotary shaker (150 rpm) at 30°C in triplicate parallels. For fatty acid composition analysis, cultivation with C/N ratio 70 as carried out in 200 mL mineral medium with one of the following N sources: (NH_4_)_2_SO_4_, KNO_3_, urea, and (NH_4_)NO_3_ with 6 mL inoculum, at 30°C on an orbital shaker (150 rpm). The cultivation was carried out for 96 h (until early stationary phase). After cultivation, the cells were centrifuged (9000 g, 10 min) and washed two times. Biomass yield was determined as dry cell weight. Biomass was frozen at −75°C and lyophilized.

### 2.3. Lipid Extraction

Lyophilized yeast was mixed with 2 mL of 0.1 mol/L Na_2_CO_3_ and the mixture was briefly ground with Ballotini glass beads (diameter 0.2 mm) in a mortar, overlaid with liquid nitrogen, and ground again. This process was repeated 3 times and 50 mL of 0.1 mol/L Na_2_CO_3_ was finally added. The crushed yeast was extracted with a chloroform-methanol mixture according to Bligh and Dyer [34]. The sample was centrifuged and the lower phase was evaporated to dryness and the lipid dry weight was determined.

### 2.4. Analysis of Fatty Acid Methylesters

The total lipids (~5 mg) were saponified overnight in 10% KOH-MeOH at room temperature. A fatty acid fraction obtained from the saponification was partitioned between alkali solution (pH 9) and diethylether to remove basic and neutral components. The aqueous phase containing fatty acids was acidified to pH 2 and extracted with hexane. The fatty acid fraction was methylated using BF_3_/MeOH (14% solution of BF_3_ from Sigma-Aldrich).

Gas chromatography-mass spectrometry of FAME was done on a GC-MS system consisting of Varian 450-GC (Varian BV, Middelburg, Netherlands), Varian 240-MS ion trap detector with electron ionization (EI), and CombiPal autosampler (CTC, USA) equipped with split/splitless injector. SP-2380 column (Supelco) (100 m, 0.25 mm ID, 0.20 *μ*m film thickness) was used for separation. The temperature program started at 60°C and was held for 1 min in splitless mode. Then, the splitter was opened and the oven was heated to 160°C at a rate of 25°C min^−1^. The second temperature ramp was up to 220°C at a rate of 1.0°C min^−1^, this temperature being maintained for 10 min. The solvent delay time was set to 8 min. The transfer line temperature was set to 280°C. Mass spectra were recorded at 3 scans s^−1^ under electron ionization at 70 eV, mass range* m/z* 50–600. FAMEs (fatty acid methyl esters) were identified according to their mass spectra and using a mixture of chemical standards obtained from Sigma-Aldrich.

Statistical analysis was performed with SigmaStat 3.5 (USA). The statistical significance of differences in mean values of the different measured parameters was calculated by one-way ANOVAs and compared with Tukey's test at the 5% level of probability.

## 3. Results

### 3.1. Lipid Production and Growth at Different C/N Ratios

**Figure 1:**
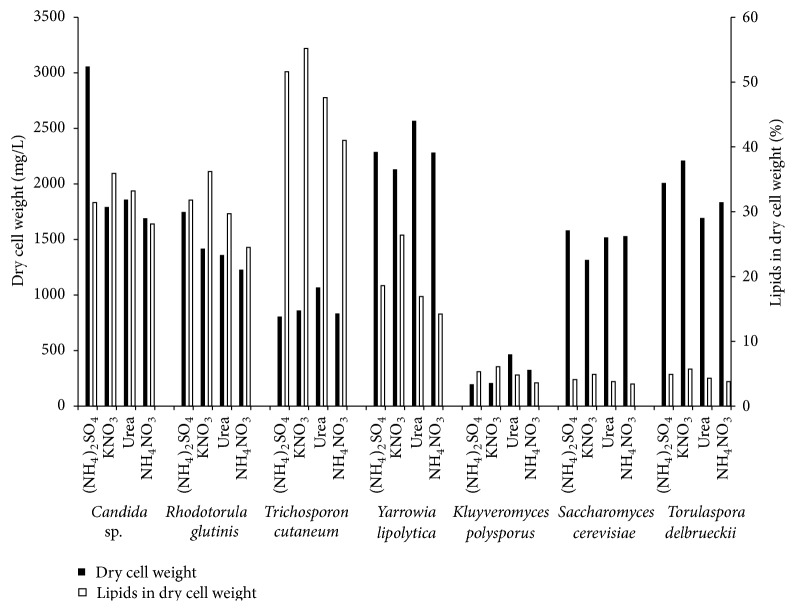
Biomass yield (black bars, dry cell weight) and lipid content in dry cell weight (white bars) of the 7 yeast strains cultured on 4 nitrogen sources at C/N ratio 70.

We compared the production of total lipid production by the seven oleaginous and nonoleaginous yeast species. The selected nonoleaginous strains are often employed in biotechnological processes and either their biomass is accessible as a byproduct of food industry or their growth requirements are well understood, allowing for easy scale-up for production in high quantities. As the microbial growth and lipid content are significantly influenced by the C/N ratio of the nutrients, we studied the yeast growth in Bioscreen C microplates at a C/N ratio of 30–90, that is, under nitrogen limitation. Glucose was used as carbon source, because although it is an expensive substrate as a pure compound, it is often used as a model substrate for the estimation of yeast production abilities. Different organic or inorganic substances can also be used as the N source: in this work, (NH_4_)_2_SO_4_, KNO_3_, urea, and (NH_4_)NO_3_. Both oleaginous and nonoleaginous yeasts showed similar growth characteristics under the same conditions of C/N ratio; that is, their lag-phase duration and biomass yield were comparable (data not shown). For all other strains, the growth in the complex YPD medium was substantially higher than in a mineral medium, whatever the C/N ratio. A C/N ratio of 70, which ensured the highest lipid content, was chosen, for comparing the lipid content of both oleaginous and nonoleaginous yeasts and detailing the fatty acid composition. Lower C/N ratios suppressed lipid accumulation and higher ratio (C/N 90) did not lead to higher lipid accumulation also. Lipid yield, expressed as the lipid content obtained from cells in a unit volume of the original medium, is dependent both on the biomass yield and on lipid content. The influence of four nitrogen sources on lipid content was studied. In cultivation with C/N ratio 70, the lipid content was in the range of 3.5–55% of dry cell weight (Figure 1), with oleaginous strains showing lipid content between 14 and 55%, decreasing in the sequence* T. cutaneum* >* Candida* sp. ≥* R. glutinis* >* Y. lipolytica*. In all four oleaginous strains, potassium nitrate supported the highest lipid content. The lipid content of nonoleaginous strains did not vary significantly in dependence on N source. The proportion of lipids in nonoleaginous strains (*S. cerevisiae, T. delbrueckii*, and* K. polysporus*) was low (3.5–6%).

When biomass yield (dry cell weight in mg/L) of individual strains was compared (Figure 1), no substantial collective preference for nitrogen source was observed among the yeasts strains. The highest overall biomass yield (~2300 mg/L) was observed in* Y. lipolytica*. Ammonium sulfate led to the highest biomass yield of* Candida* sp. and* R. glutinis*, and urea as an organic N source increased the biomass content in cultivation of* T. cutaneum* and* Y. lipolytica*. Under studied conditions, nonoleaginous strains did not produce lipids or biomass in high amount.

When both biomass yield and lipid content were compared,* Candida* sp. and* R. glutinis* displayed a high ratio of lipid/biomass production, 30 and 40%, respectively, whereas the lipid/biomass ratio in* Y. lipolytica* was a mere 15–25% depending on N source. The lipid content in cells was found to be dependent on the type of microorganism and its genus. For instance, the lipid content of* T. cutaneum* was three times higher than the lipid content of* Y. lipolytica* (20%) under the same cultivation conditions, although both strains are oleaginous.* T. cutaneum* that produced a high amount of lipids (55%), but biomass yield was low (approx. 1 g/L). On the other hand,* Candida* sp. produced 40% lipids in biomass, but the biomass yield was twice as high compared to* T. cutaneum*.

### 3.2. Fatty Acid Composition and Profile

Table 1 shows the total lipid content and saturated, monounsaturated, and polyunsaturated fatty acid profile of all studied strains in dependence on the N source used (C/N ratio 70). Major fatty acids were oleic (18 : 1), palmitic (16 : 0), and linoleic (18 : 2) acids, while stearic (18 : 0), arachidic (20 : 0), and palmitoleic (16 : 1) acids were present in lesser amounts. The fatty acid composition in all seven strains is similar to the fatty acid composition found in plant oils and might be therefore utilized in biodiesel production.* S. cerevisiae* biomass is produced in high amounts in biotechnological applications, with very low lipid content (less than 10%) of which palmitoleic acid (16 : 1) comprises 40%.* K. polysporus* produced up to 60% of palmitoleic acid, but the total lipid and biomass yield was low in comparison with other strains (Figure 1).

The strains that showed a very high content of unsaturated fatty acids were* Y. lipolytica* (84%)*, Candida* sp. (80%),* R. glutinis* (79%)*, T. cutaneum* (79%)*, K. polysporus* (89%),* S. cerevisiae* (81%), and* T. delbrueckii* (67%) (relative to total FA content). The highest proportions of unsaturated fatty acids were obtained in cultivation on ammonium sulfate. If the biomass yield of the strains cultivated on different N sources was compared, urea will be seen to lead to the highest biomass content in* T. cutaneum, Y. lipolytica*, and* K. polysporus* while ammonium sulfate supported the highest biomass production in* S. cerevisiae, Candida* sp., and* R. glutinis*. Potassium nitrate was found to be the most appropriate nitrogen source for biomass production in* T. delbrueckii*.

Very different results were found when considering lipid content (%) in dependence on nitrogen source. Here cultivation on potassium nitrate leads to the highest lipid contents in all studied strains. The second most suitable N source is ammonium sulfate (for 6 strains, safe for* Candida* sp.). When considering the lipid content relative to unit medium volume and biomass, ammonium sulfate appears to be the most suitable N source for 3 strains (*Candida* sp.,* R. glutinis,* and* S. cerevisiae*), urea for* T. cutaneum *and* K. polysporus*, and potassium nitrate for* T. delbrueckii* and* Y. lipolytica.*

### 3.3. Palmitoleic and Linoleic Acid Content

**Figure 2:**
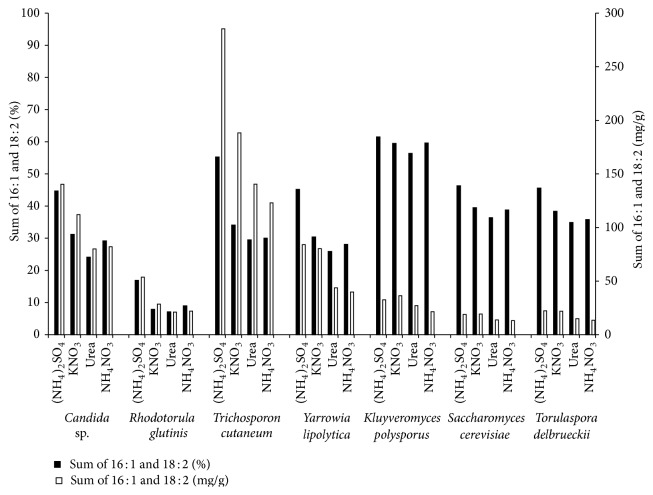
Content of palmitoleic and linoleic acid in total lipids (%, black bars) and the yield of the acids per dry cell weight (mg/g dw, white bars) of the 7 yeast strains cultured on 4 nitrogen sources at C/N ratio 70.

Figure 2 depicts the combined content of the two unsaturated dietary fatty acids of interest, palmitoleic and linoleic acid, as a ratio of total fatty acids (%) and in mg per dry cell weight in all seven studied strains cultivated with four different nitrogen sources. The figure shows that the content and yield of the studied fatty acids are dependent on the yeast strain and cultivation conditions. The nonoleaginous strains showed higher average proportion of both unsaturated fatty acids, in* K. polysporus* in all studied conditions over 50%,* S. cerevisiae* and* T. delbrueckii* over 40% with minor preference for the ammonium sulfate nitrogen source. However, in oleaginous yeast,* T. cutaneum* has been found to have the highest yield—the sum of palmitoleic and linoleic acid per dry cell weight—up to 285 mg/g (dry cell weight) when ammonium sulfate was used as the nitrogen source. That is more than 30% increase in comparison with the second highest yield per dry cell weight (*T. cutaneum* with potassium nitrate) and twice as high as the next third highest yield per dry cell weight (*Candida* sp. with ammonium sulfate and* T. cutaneum* with urea).

## 4. Discussion

At present, microbial oils are studied as an alternative to many traditional raw materials and human food and health produce [1]. Either for biofuel utilization or for health supplements, yeasts, especially oleaginous ones like* Y. lipolytica, Cryptococcus curvatus, R. glutinis*, and* Trichosporon* species, are intensively investigated [10].

The ability of oleaginous yeast to accumulate lipids or produce lipids of specific composition is dependent on many factors such as carbon source, nutrient availability, cultivation temperature, pH, inoculum size, trace element content, and cultivation time. In this work, glucose was chosen as a substrate; however, many other starting materials are studied, besides saccharides, such as lactose or sucrose [35] that can be also exploited as volatile fatty acids [21] or industrial fat derivatives composed of saturated free fatty acids [18]. The utilization of VFA or industrial fat influences the lipid mechanism synthesis, which is then different than that on glucose [19, 20].

Nitrogen source shortage has a significant effect upon the FA composition of microbial lipids. Recent investigations on yeast and algae pointed out a close relationship between nitrogen starvation, autophagy, and production of storage lipid, especially triacylglycerols. In yeasts, nutrient starvation induces a high level of autophagy [36–38]. Li et al. [39] showed that nitrogen starvation is indeed a universal stimulus of autophagy in yeast. Under nitrogen starvation, yeast cells continue to consume glucose, downregulate fermentation, and produce increased amounts of storage lipid, largely in the form of lipid droplets. A crucial factor for autophagosome biogenesis was found to be triacylglycerol synthesis. Boyle et al. [40] investigated the factors that trigger the production of triacylglycerols in algae and discovered the genes responsible for triacylglycerol (TAG) production in* Chlamydomonas* and for the regulatory components that activate the pathway. Several genes encoding acyltransferases (diacylglycerol acyltransferase and/or phospholipid-diacylglycerol acyltransferase) were found to be induced by nitrogen starvation and are likely to have a role in TAG accumulation conditions.

The key factors used in our work were based on previous laboratory results and the information acquired from the literature: temperature 28°C, pH 6.0, cultivation time 96 h, and 3% inoculum [31, 41–44]. Among the most important factors in lipid accumulation is low availability of nutrients, most commonly nitrogen source [19].

Under such conditions, it can be proved that higher C/N ratio in the medium leads to higher lipid accumulation. This fact is dependent on the studied yeast species and the cultivation conditions (substrate type, concentration, and cultivation setup); all of these factors define the existence and value of the optimum C/N ratio for lipid accumulation and are usually determined experimentally [35]. Our findings led to the conclusion that, for the studied yeast strains and selected cultivation conditions, the lipid content at C/N ratio 70 was higher than at C/N 30 [17]. Some authors give a C/N ratio of approximately 100 as the best for lipid production but the difference in cultivation conditions is the determining factor [2, 5, 45]. When lipidic substrates are used, high C/N ratios for lipid synthesis are preferred by many yeasts [18]. For continuous cultivation, a mathematical model was created and the effect of C/N ratio and dilution rates on lipid production by oleaginous yeasts has been evaluated by this model. For these conditions, authors state that lipid yield increased gradually with increasing C/N ratios. For continuous cultivation, lipid yield and lipid production rate can be calculated at any C/N ratio of the growth medium and optimum operation conditions can be predicted for the production of microbial lipids by using this modelling [46]. In our work, C/N ratio 70 was found to lead to the highest lipid accumulation, and C/N ratio 90 led to a decrease in lipid content. Braunwald et al. [47] reported that higher initial glucose concentration does not lead to further lipid accumulation. Similar results were reported in other studies [18, 48], and our study therefore focused on increasing the lipid content by using different types of nitrogen sources and yeast strains rather than employing higher initial glucose concentration, which was used as a model carbon source intended for the establishment of the potential of studied yeasts for lipid accumulation and unsaturated fatty acid production. For industrial-scale production, scale-up would have to be performed and selection of carbon source from the wide array of saccharide waste products would be convenient for better economical balance of the process and mathematical modelling might be exploited [46].

The aim of this work was to interconnect the nitrogen source influence on the total lipid accumulation and the production of dietary unsaturated fatty acids (palmitoleic and linoleic), rather than for biodiesel production. Feasibility studies state that the cultivation of yeasts for microbial biomass and subsequent biodiesel production are substantially limited by the cost required by the cultivation process [12].

There are only a few studies comparing the influence of C/N ratio and the nitrogen source type on biomass yield. Our study is unique in evaluating the influence of these two parameters on both oleaginous and nonoleaginous yeasts. The results suggest that the biomass yield is determined by the nitrogen source type while a change in C/N ratio (with the same nitrogen source) does not influence growth. Negligible changes in biomass amount caused by changing C/N ratio were also reported by Wiebe et al. [11], Braunwald et al. [47], and Galafassi et al. [49]. A study by Jadhav et al. [50] on* Y. lipolytica* and* Lipomyces slipover* reported an influence of nitrogen type, but without specifying the influence of C/N ratio, as did Liu et al. [42].

Similarly, as reported by Turcotte and Kosaric [51] on* Rhodosporidium toruloides*, we found no preferential nitrogen source for biomass yield alone.

Our data show that the ability to accumulate lipids is genus-dependent (see also the study by Sitepu et al. [8]). In all seven strains, maximum lipid production (%) was observed on potassium nitrate; similar results were reported by Jadhav et al. [50] who studied* Y. lipolytica.* The maximum lipid content per produced cell biomass (Figure 1) of our yeast strains was as follows:* T. cutaneum* 55.2%,* R. glutinis* 36.2%*, Candida* sp. 35.9%*, Y. lipolytica* 26.4%*, K. polysporus* 6.1%*, T. delbrueckii* 5.7%, and* S. cerevisiae* 4.9%. The results observed for* Candida* sp. and* R. glutinis* are consistent with the data reported in the literature [52].

Lipid yield on different nitrogen substrates is also genus-dependent [7]. In our study, lipid production after 96-hour cultivation reached 0.30–0.96 g/L medium. The highest values were displayed by* Candida* sp. Nonoleaginous species produced lipids in lesser amounts: 0.01 (*S. cerevisiae*) and 0.13 g/L (*T. delbrueckii*). Low values of lipid accumulation might be the result of insufficient aeration [53] or a rapid pH drop, caused by acidifying effect of ammonium uptake by the cell at early stages of cultivation, reported to be detrimental to lipid production [54]. To our knowledge, there are no studies comparing a series of different yeast strains as to the suitability of nitrogen sources for lipid yield. In our study, urea was found to be the most suitable nitrogen source for lipid yield in the nonoleaginous* K. polysporus* and the oleaginous* T. cutaneum*. The same result was observed by Zhu et al. [31] for* T. fermentans* and Evans et al. [55] for* R. toruloides*. We found ammonium sulfate as the most suitable nitrogen source for lipid yield in oleaginous* Candida* sp.*, R. glutinis*, and* S. cerevisiae*. It was also found to ensure the highest lipid yield in* Lipomyces starkeyi* [42] whereas da Rosa et al. [56] mentioned ammonium sulfate as the least appropriate nitrogen source for lipid yield in* C. zeylanoides*.

On comparing the results, ammonium sulfate is seen to be the most appropriate nitrogen source for the highest biomass production, lipid content in biomass dry cell weight, and lipid yield (see also [57, 58]).

Ammonium is the main nitrogen source in AFEX-pretreated corn stover hydrolysate [59] which might be therefore suitable for cultivating our strains when scale-up is considered.

The fatty acid profile determined in this study displayed preferential synthesis of unsaturated fatty acids (palmitoleic acid (16 : 1), oleic acid (18 : 1), and linoleic acid (18 : 2)) in both oleaginous and nonoleaginous species. The content of these fatty acids comprised up to 60–90% of total FA. These data are consistent with published results [41, 53]. The unsaturated fatty acid content increases with cultivation duration and under nitrogen starvation.

Yeasts that are able to produce dietary fatty acids (palmitoleic and linoleic) in high amount might be utilized for biotechnological production for nutritional use [7].

The content of linoleic acid (18 : 2), an essential omega-6 fatty acid, is also influenced by the composition of cultivation medium and cultivation time. In our study, its production in all studied strains significantly increased when ammonium sulfate was used as N source. The oleaginous yeast* T. cutaneum* has been found as the most promising for its high yield of palmitoleic and linoleic acid per dry cell weight, up to 285 mg/g per dry cell weight when ammonium sulfate was used as the nitrogen source. Also, the highest content of linoleic acid (18 : 2) was observed in* T. cutaneum* (53.4%).

The nonoleaginous yeasts showed preferential production of palmitoleic and linoleic acid, which might be advantageously utilized in biomass byproduct utilization. Although these yeasts did not show under studied conditions high biomass yield, their production of unsaturated fatty acids did not vary significantly under different cultivation conditions. This might be taken advantage of in the processing of biomass waste product of the brewing or wine industry. Such biomass is easily obtainable with low cost and the unsaturated fatty acid might be extracted from this material with positive economical balance.

For oleaginous species, optimization of lipid production and higher biomass and lipid yield in terms of total expenses might be achieved by modifying cultivation configuration (batch or fed-batch cultivation) in a bioreactor or utilization of waste materials [31, 44].

## 5. Conclusion

All studied oleaginous and nonoleaginous yeast species (*Yarrowia lipolytica, Rhodotorula glutinis, Candida *sp.,* Trichosporon cutaneum, Torulaspora delbrueckii, Kluyveromyces polysporus, *and* Saccharomyces cerevisiae*) have been observed to ascertain their ability to produce specific unsaturated fatty acids. Oleaginous strains with high lipid content and high unsaturated fatty acids yield might be utilized for specific production, with* T. cutaneum* as the most promising candidate. Although the nonoleaginous species lipid content is lower, their advantageous property is their high content of monounsaturated fatty acids under wide array of cultivation conditions and the possibility of their acquisition as food industry waste products. The best nitrogen source for lipid accumulation was potassium nitrate, followed by ammonium sulfate, which is an ideal nitrogen source supporting sufficient biomass growth with concomitantly increased lipid accumulation in both oleaginous and nonoleaginous species. Using this optimum N source has a higher importance for the proper biomass-lipids ratio than the change of the C/N ratio in the range under study.

## Figures and Tables

**Table 1:** Proportion (%) of fatty acids and total mono- and polyunsaturated fatty acids in the 7 yeast strains cultivated with 4 nitrogen sources.

Strain	N source C/N = 70	16 : 0	16 : 1	18 : 0	18 : 1	18 : 2	20 : 0	Σ Sat	Σ Mono	Σ Poly
*Candida sp.*	(NH_4_)_2_SO_4_	13.8	7.9	3.4	36.0	36.8	2.1	19.3	43.9	36.8
	KNO_3_	17.3	9.4	4.5	43.4	21.8	3.6	25.4	52.8	21.8
	Urea	18.1	10.9	4.4	46.7	13.2	6.7	29.2	57.6	13.2
	NH_4_NO_3_	16.7	12.3	4.5	45.6	16.9	4.0	25.2	57.9	16.9
*Rhodotorula glutinis*	(NH_4_)_2_SO_4_	13.1	1.3	6.9	61.7	15.6	1.4	21.4	63.0	15.6
	KNO_3_	17.3	1.6	7.4	65.7	6.3	1.7	26.4	67.3	6.3
	Urea	16.8	1.7	8.0	65.0	5.4	3.1	27.9	66.7	5.4
	NH_4_NO_3_	16.0	1.5	9.1	64.4	7.5	1.5	26.6	65.9	7.5
*Trichosporum cutaneum*	(NH_4_)_2_SO_4_	16.0	1.9	3.2	23.9	53.4	1.6	20.8	25.8	53.4
	KNO_3_	19.4	2.3	4.4	38.9	31.8	3.2	27.0	41.2	31.8
	Urea	20.5	2.6	5.8	38.2	26.9	6.0	32.3	40.8	26.9
	NH_4_NO_3_	20.4	2.6	5.1	38.9	27.4	5.6	31.1	41.5	27.4
*Yarrowia lipolytica*	(NH_4_)_2_SO_4_	10.3	9.4	2.3	38.7	35.8	3.5	16.1	48.1	35.8
	KNO_3_	14.5	10.6	4.1	45.7	19.8	5.3	23.9	56.3	19.8
	Urea	15.3	11.2	3.7	46.0	14.7	9.1	28.1	57.2	14.7
	NH_4_NO_3_	14.6	11.3	4.0	47.9	16.8	5.4	24.0	59.2	16.8
*Kluyveromyces polysporus*	(NH_4_)_2_SO_4_	8.6	57.2	1.2	27.6	4.3	1.1	10.9	84.8	4.3
	KNO_3_	9.2	58.0	1.5	28.1	1.5	1.7	12.4	86.1	1.5
	Urea	9.6	55.3	1.8	28.9	1.1	3.3	14.7	84.2	1.1
	NH_4_NO_3_	9.0	58.1	1.5	28.6	1.5	1.3	11.8	86.7	1.5
*Saccharomyces cerevisiae*	(NH_4_)_2_SO_4_	11.5	38.9	3.9	35.2	7.4	3.1	18.5	74.1	7.4
	KNO_3_	14.1	38.5	4.0	39.1	1.0	3.3	21.4	77.6	1.0
	Urea	12.4	35.5	5.7	38.7	0.9	6.8	24.9	74.2	0.9
	NH_4_NO_3_	13.2	37.7	3.9	41.0	1.1	3.1	20.2	78.7	1.1
*Torulaspora delbrueckii*	(NH_4_)_2_SO_4_	21.4	18.4	3.0	21.9	27.2	8.1	32.5	40.3	27.2
	KNO_3_	26.2	22.8	4.1	26.0	15.6	5.3	35.6	48.8	15.6
	Urea	24.3	21.7	4.5	27.1	13.2	9.2	38.0	48.8	13.2
	NH_4_NO_3_	26.6	21.7	4.1	28.2	14.1	5.3	36.0	49.9	14.1
